# Introductory Lectures

**Published:** 1860-09

**Authors:** 


Introductory Lectures.—The different medical schools of this city will hereafter deliver only one general introductory lecture at the opening of their respective sessions. This year the lecture will come off on Monday, the eighth of October, the didactic course commencing on the following day.

